# Mechanically Tunable Extracellular Matrix of Genipin Crosslinked Collagen and Its Effect on Endothelial Function

**DOI:** 10.3390/app12052401

**Published:** 2022-02-25

**Authors:** Jacob Robinson, Teal Russell, Zhigang Xu, Yeoheung Yun

**Affiliations:** 1 FIT BEST Laboratory, Department of Chemical, Biological, and Bio Engineering, North Carolina A&T State University, Greensboro, NC 27411, USA; 2 Department of Mechanical Engineering, North Carolina A&T State University, Greensboro, NC 27411, USA

**Keywords:** collagen-I, genipin, endothelial, TEER

## Abstract

Mechanical rigidity of a matrix, to which cells adhere, plays a significant role in regulating phenotypic cellular behaviors such as spreading and junction formation because vascular cells sense and respond to changes in their mechanical environment. Controlling mechanical properties of extracellular matrix by using a crosslinker is important for cell and tissue mechanobiology. In this paper, we explored genipin, a natural plant extract, to crosslink collagen-I in order to enhance mechanical properties with low cytotoxicity. We characterized the effects of genipin concentration on the mechanical properties, color change, degradation, structure, cell viability, and endothelial function such as transendothelial electrical resistance (TEER). Through the analysis of both material properties and endothelial response, it was found that genipin-based glycation caused an increase in viscoelastic moduli in collagen hydrogels, as well as increased fiber density in their structural morphology. Endothelial cells were found to form better barriers, express higher levels of tight junction proteins, and exhibit better adhesion on stiffer matrices.

## 1. Introduction

Hydrogels created using natural biomaterials such as collagen have been documented to provide an appropriate extracellular matrix (ECM) mimetic for endothelial cell culture, as opposed to culturing in polystyrene plates [1]. Type I collagen has been commonly used for its ability to support cell adhesion and signaling [2]. However, collagen gels suffer from poor mechanical strength, causing adverse phenotypes in endothelial cells which utilize mechanotransduction to enact various cell processes, and produce appropriate cell morphologies and signals [3]. This plays a key role in the exploration of disease pathologies, particularly of those associated with aging, such as Alzheimer’s disease (AD). This is due to the progressive glycation and mechanical change of vascular ECM in the brain as one ages, specifically pertaining to the ECM of the blood–brain barrier (BBB), which is responsible for protecting the sensitive brain environment from harmful molecules [4].

Crosslinking the ECMs through glycation poses another obstacle via potential cytotoxicity [5]. Genipin is commonly used to crosslink collagen gels—an iridoid compound derived from the fruit of Gardenia jasminoides—which results in minimal cytotoxicity [6,7]. However, this inherent cytotoxicity effectuates a mutual exclusivity between cellular viability and gel strength, especially with higher concentrations of genipin [8].

Few studies document methods of striking a balance between increasing gel strength using genipin, while also maintaining cellular viability. The objective of this study is therefore multifold: to create cell-viable hydrogels of variable mechanical strength using genipin; to see how the mechanical properties of these gels correlate with structural morphology; and to monitor the endothelial behavior and adhesion of the cells grown on these gels. These findings provide a way to produce tunable collagen hydrogels which: allow for resultant tunability of endothelial response and signaling; can be used to simulate the glycation of the brain ECM; and in further studies allow for more accurate modeling of age-related diseases such as AD.

## 2. Materials and Methods

### Gel Preparation:

Solubilized collagen type I was obtained from Advanced BioMatrix, USA, with a stock concentration of 3.1 mg/mL. On ice, the solubilized collagen was mixed with 1 M HEPES buffer and 10X phosphate buffered saline (PBS), and diluted with deionized water, in order to reach final concentrations of 2.5 mg/mL collagen, 25 mM HEPES, and 1X PBS. Using a 0.5 M NaOH solution, the gel solution was titrated to a pH of 7.4. This base gel solution was used in both the control and two treatments. This neutralized collagen solution was split between use for material characterization and cell culture.

In order to conserve the amount of genipin available, the treatment for these gels was split between pre-glycation and post-glycation, the difference between the two being that pre-glycated gels are crosslinked while still in solution and before polymerization, whereas post-glycation gels are crosslinked after being polymerized. To form the post-glycated collagen gels, genipin solutions were made by dissolving genipin in 10 mL 1X PBS to concentrations of 1 mM and 10 mM. After the base gels were polymerized, they were incubated in the respective genipin solution for 24 h to maximize glycation. To form the pre-glycated gels, solid genipin was added to the collagen solution and mixed thoroughly to ensure even distribution of genipin, making 13 and 22 mM gels. The solutions were then added to the wells and incubated at 37 °C, 5% CO_2_. To make a higher concentration post-glycation solution requires more genipin than was available at the time of this experiment, so pre-glycation was used for higher concentration gels. A test sample of four 10 mM gels was created through both pre- and post-glycation and were tested to confirm the negligible difference in modulus value between glycation method.

To wash out residual genipin, a series of washing steps was introduced. These steps involved four 60-min washes in 3% glycine, three 90-min washes in 70% ethanol, and four 30-min 1X PBS washes. The gels were washed on a rocker at room temperature, with two rinses with deionized water between each wash. After washing, the gels were incubated with EGM-2 medium at 37 °C, 5% CO_2_ for one hour prior to seeding the cells.

### Rheological characterization:

All rheological measurements were performed on a Discovery Hybrid Rheometer (DHR-2, TA Instruments, DE, USA). Separate collagen gels were prepared as described above and used for rheological purposes only. Samples were loaded on parallel plate geometries, and an oscillatory time sweep was used. This compressed the gels under 10% strain and an angular frequency of 1 Hz. The time sweep was run for one minute, acquiring a total of 10 measurements. Storage modulus and loss modulus were measured. Storage modulus is an elastic portion of collagen gels and loss modulus is a viscous portion of collagen gels.

### Advanced Glycation End-product (AGE) Fluorescence:

To detect the presence of AGEs, the gels were prepared and polymerized in plastic cuvettes before being placed in a BioMate 3S UV-Visible Spectrophotometer (Thermo Fisher, Waltham, MA, USA). Using an excitation wavelength of 365 nm, an absorbance scan was run on the spectrophotometer using light beams spanning wavelengths from 200 to 400 nanometers (nm), using a blank cuvette as a baseline. Each sample was scanned in triplicate to ensure accuracy of the measurements. Data were saved in tabular form in Microsoft Excel and then recompiled into a graph using GraphPad Prism software.

### Scanning Electron Microscopy (SEM):

In order to acquire proper scaffolds for SEM visualization, the gels were prepared in a 24-well plate and polymerized. They were then frozen at −20 °C for three hours, before being lyophilized using a FreeZone 2.5 Plus freezedrier (LabConco, USA) for 14 h. To prepare the gels for imaging, a piece of each gel was cut and placed onto the SEM stage. The stage was then placed into a Cressington sputter coater (Watford WD England, UK), where the samples were placed under a vacuum using argon gas. Samples were then sputter-coated in a mixture of gold and palladium. The samples were then imaged on a Hitachi SU8000 field emission scanning electron microscope under a vacuum.

### Cell Culture:

The endothelial cells were pooled donor human umbilical vein endothelial cells (HUVECs, Lonza) that were pre-screened for angiogenesis. Endothelial basal medium-2 (EBM-2) was also obtained from Lonza, USA, along with the accompanying BulletKit. Cells were thawed and diluted at a ratio of 1:10 with fresh EGM-2 medium. This cell suspension was then transferred to a T75 flask and placed in a 37 °C, 5% CO_2_ incubator. The medium was refreshed after the first 24 h and then every 48 h. HUVECs typically reached confluency in 3–5 days. All cellular experiments were conducted after 3 days of growth.

### Proliferation and Viability Assay:

In a 24-well plate, 15 collagen scaffolds were created with concentrations of 0, 1, 10, 13, and 22 mM genipin. Cells were seeded onto these scaffolds at a density of 60,000 cells per scaffold. Pictures of the cell cultures were taken daily on an EVOS FL digital inverted microscope (Thermo Fisher, Waltham, MA, USA). The number of cells per picture was then quantified using ImageJ (Mad City Labs, Madison, WI, USA) (1.53). The pictures were edited through the subtraction of the image background and thresholding. After analyzing and counting the cells in each picture, the quantitative data were saved into a spreadsheet. This data were then imported into GraphPad Prism software to see the rate of cell proliferation and total cell counts among substrates.

The LIVE/DEAD Viability/Cytotoxicity kit (Thermo Fisher, USA) was used to visualize cell viability across the collagen scaffolds. After conducting the proliferation assay, cells were washed of medium with D-PBS. After washing the cells, the assay solution, consisting of 4 μM ethidium homodimer 1 (EthD-1) and 2 μM calcein-am in D-PBS, was dispensed on top of each scaffold, ensuring that all cells were covered. The cells were incubated at room temperature for 45 min and then imaged under the microscope. ImageJ was used to quantify the fluorescence of living cells after using the cytotoxicity kit. Using pictures taken on the microscope, analysis was run—specifically measuring the area and integrated intensity—on both the cells and the background of the image. This was repeated three times per sample, and data were compiled into a spreadsheet. The corrected total cell fluorescence (*CTCF*) was calculated using (1)

(1) $$CTCF=D_{i}-\left(A_{\text{cell}}*F_{bg}\right)$$

where *D*_*i*_ is the integrated density, *A*_*cell*_ is the area of the selected cell(s), and *F*_*bg*_ is the mean background fluorescence. All fluorescence measurements, including *CTCF*, were calculated in relative fluorescence units (RFUs).

### Transendothelial electrical resistance (TEER):

Transendothelial electrical resistance (TEER) was used as a nondestructive way to measure barrier integrity. In a 12 Transwell plate, 10-well inserts were coated in control, 1, 10, 13, and 22 mM gel solutions, and polymerized, resulting in two gels per treatment. After washing, a baseline resistance value was taken using an EVOM 2 Voltohmmeter. Cells were seeded at a density of 56,000 cells per scaffold and cultured for 3 days, with their resistance being measured daily. TEER was calculated using (2) and (3) to correct for any resistance due to the substrate.


(2) $$R_{\text{tissue}}=\left(R_{\text{total}}-R_{\text{blank}}\right)$$

(3) $$TEER_{\text{reported}}=R_{\text{tissue}}\left(\Omega \right)*M_{\text{tissue}}\left(cm^{2}\right)$$

### Cell morphology Measurement:

Collagen scaffolds were created in the wells of a 96-well plate at concentrations of 0, 1, 10, 13, and 22 mM genipin. After washing the scaffolds, cells were seeded at a density of 20,000 cells per scaffold and cultured for 3 days. Staining for claudin-5 was performed by fixing and permeabilizing the cells in a 4% paraformaldehyde solution and a 0.1% Triton X-100 permeabilization buffer, respectively. The cells were then blocked using a 3% fetal bovine serum. The primary antibody solution was made by diluting 250 ug/mL antibodies for claudin-5 to their working concentration of 5μg/mL using the blocking solution. The cells were incubated in the primary antibody solution overnight at 4 °C, wrapped in foil on a rocker. The secondary antibody solution was made by diluting 10,000 μg/mL Hoechst stain to 2 μg/mL in the blocking solution. Since the anti-claudin-5 primary antibody was unconjugated, a 2000 ug/mL secondary goat anti-rabbit antibody that was conjugated to a fluorescent tag was used at a working concentration of 4 ug/mL. The cells were washed three times with 1X PBS, before being incubated with the secondary antibody solution for 2 h on a rocker and wrapped in foil. The antibody solution was removed, and the cells were washed three times with 1X PBS. Wells were filled with 100 uL PBS each and visualized under a two-photon confocal microscope (Zeiss 710).

### Statistics:

All statistical analyses were conducted using GraphPad prism software. A one-way ANOVA test was used to determine difference among means, while a pairwise multiple-comparison test was used to determine differences between means. A *p*-value < 0.05 will be considered statistically significant.

## 3. Results and Discussion

### Genipin-glycated collagen:

**Figure 1.**
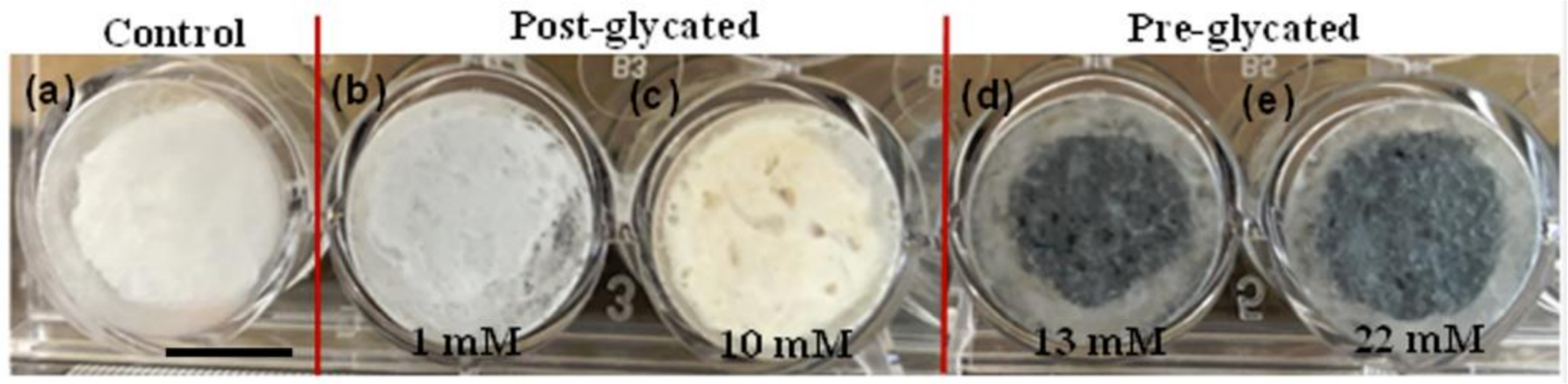
Gross morphology of lyophilized tissue-engineered cylindrical constructs with genipincross-linked collagen: (**a**) control, (**b**,**c**) post-glycated (1 mM and 10 mM), and (**d**,**e**) pre-glycated (13 mM and 22 mM) samples in 24 well plate (well diameter is 14 mm). Scale bar = 6.15 mm.

The glycation of collagen using genipin resulted in a color change between the control and glycated gels (Figure 1). In addition, the treatment of gels between pre- and post-glycation also resulted in a color change. The control collagen gels were uniformly a translucent white color (Figure 1a), while glycated gels turned blue and brown when they were pre- or post-glycated, respectively (Figure 1b–e). Pre-glycated samples were consistently darker than post-glycated samples, pointing to the possibility that pH has an effect on the color that genipin turns. In the case of pre-glycated gels, genipin was present when the scaffolds were neutralized with 0.5 M NaOH, while the post-glycated gels were already polymerized and no pH adjustment took place.

Color change of genipin-crosslinked gel has been previously reported [6,8]. Genipin is often used as a blue dye in the food industry, and has been reported to change color depending on the amino acid with which it reacts [6]. Other pigments derived from genipin glycation were blue-green, green, purple, and black [9]. Browning is a common effect of the Maillard reaction by which glycation processes in general take place [9].

### Rheology:

**Figure 2.**
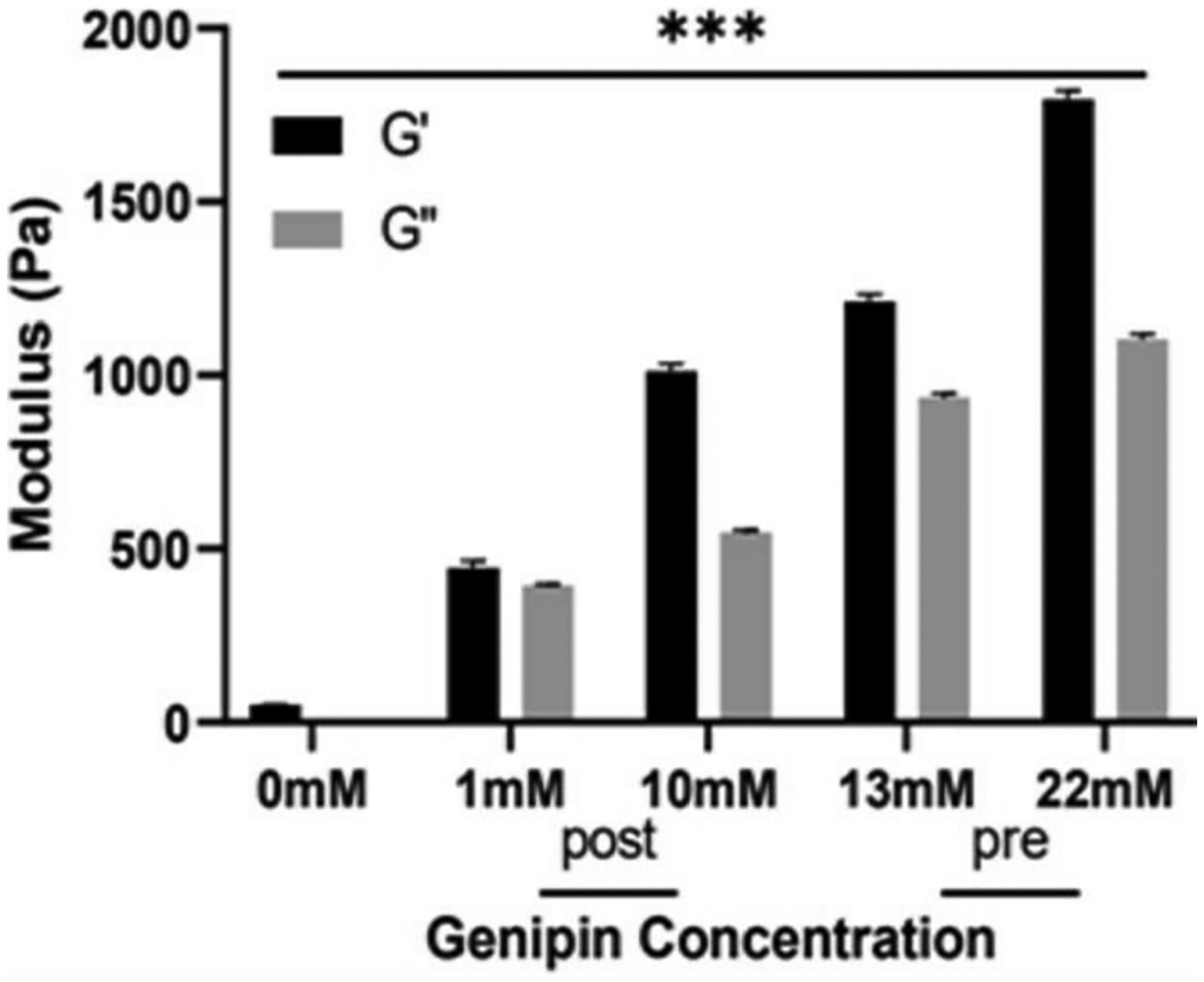
Storage (G’) and loss (G”) moduli of control, pre- and post-glycated collagen scaffolds (*** *p* < 0.0001 between all samples).

In terms of mechanical testing, glycation with genipin increased the physical properties of the collagen gels across all treatments. As shown in Figure 2, both the storage (G’) and loss modulus (G”) values increased with genipin concentration, with the G’ of the 22 mM gel exceeding the G’ of the control gel nearly 37 times. Using Tukey’s multiple pairwise comparison test, G’ of all treatments was found to be significantly different (*p* < 0.0001) from each other, as well as the G” between treatments.

In terms of the mechanical properties of the glycated gels, similar trends of moduli increase have been reported [5]. After a two hour incubation period, Sundararaghavan reported 0 mM, 1 mM, and 10 mM genipin gels having storage moduli of approximately 80, 90, and 600 Pa, respectively. Similarly, Výborný et al., (2019) created genipin gels in comparison to crosslinking agent, EDC [10]. The genipin-crosslinked gels were developed using genipin dissolved in a solution of DMSO and PBS. This resulted in 10 mM gels with a storage modulus of 100 Pa when tested using a frequency sweep. In the future, further characterization is needed, including degraded fractions and mechanical stability as tissue grows in the scaffold.

### AGE Fluorescence:

**Figure 3.**
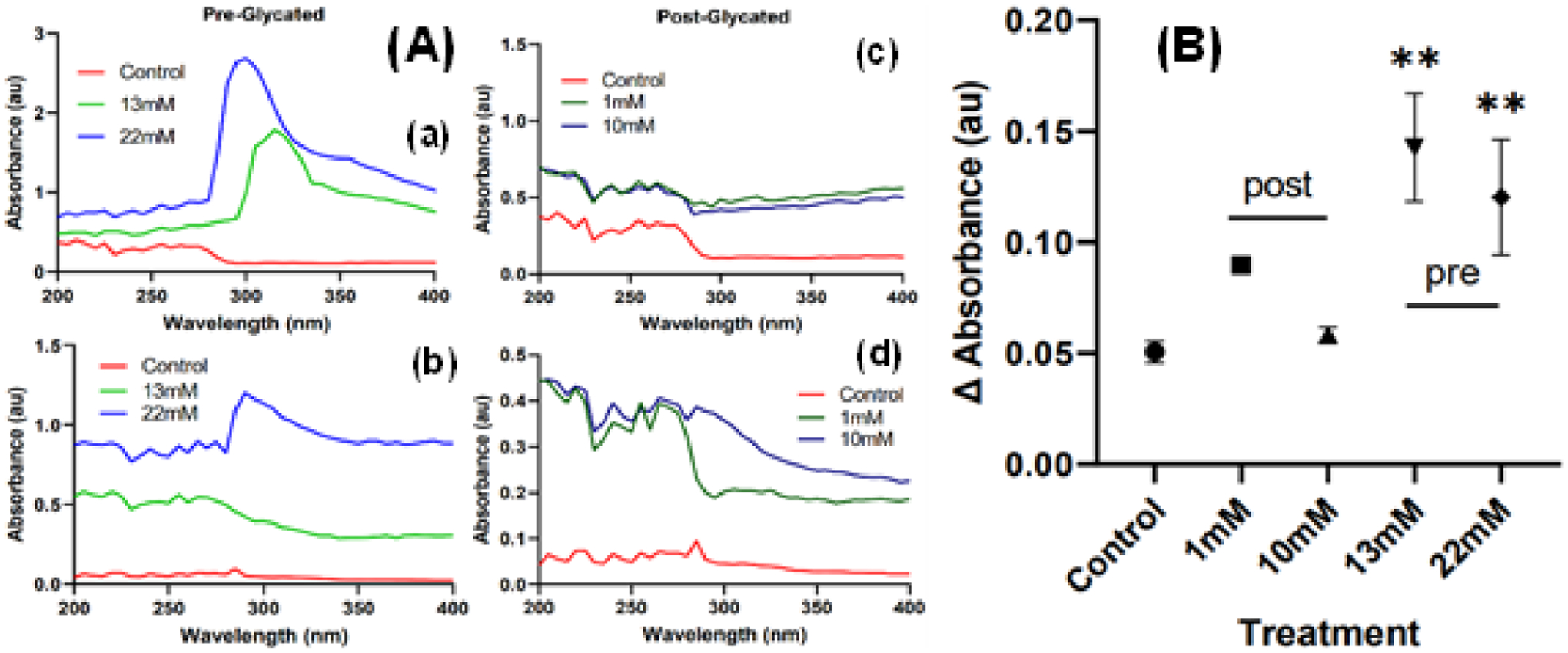
(**A**) Absorbance measurements of collagen scaffolds compared to pre-(**a**,**b**) and post-glycated scaffolds (**c**,**d**). Measurements were taken on day 1 (**top**, (**a**,**c**)) and day 3 (**bottom**, (**b**,**d**)). Absorbance values were obtained in triplicate and averaged. (**B**) Total degradation for each scaffold measured in change in average absorbance between Day 1 and Day 3. (** = *p* < 0.001).

The presence of AGEs in the collagen scaffolds increased the level of absorbance within the glycated gels when compared to the control (Figure 3). The post-glycated gels followed a near-identical pattern of absorbance with the control gel, albeit at higher absorbance values. However, for the pre-glycated gels, new absorbance peaks formed at around 300–320 nm for both the 13 and 22 mM samples. The decay of absorbance in glycated gels was also measured, as higher levels of glycation correlate with higher rates of degradation [11–13]. After measuring the total degradation in absorbance between days 1 and 3 of the gels, it was found that the pre-glycated gels experienced a significantly higher degradation rate than the control gels (Figure 3). However, while all glycated gels had higher absorbance rates due to the presence of AGEs, post-glycated gels were found to have similar rates of degradation as the control gel.

The difference in absorbance spectra between pre- and post-glycated scaffolds could potentially be from the pattern of color change exhibited in the gels. While the post-glycated gels maintained a yellow-brown hue, the pre-glycated gels often turned darker colors. Another main constituent of collagen helices is the amino acid proline, which has been reported to elicit a black color when reacting with genipin [14]. The action of genipin upon proline residues on the collagen could result in AGEs unique to pre-glycation, which would explain the formation of new peaks in the absorbance spectra. This exact mechanism is as of yet unknown, but may be due to genipin being dispersed throughout the still-solubilized gel solution, rather than acting upon the surface of already-polymerized gels.

### Scanning Electron Microscopy (SEM):

**Figure 4.**
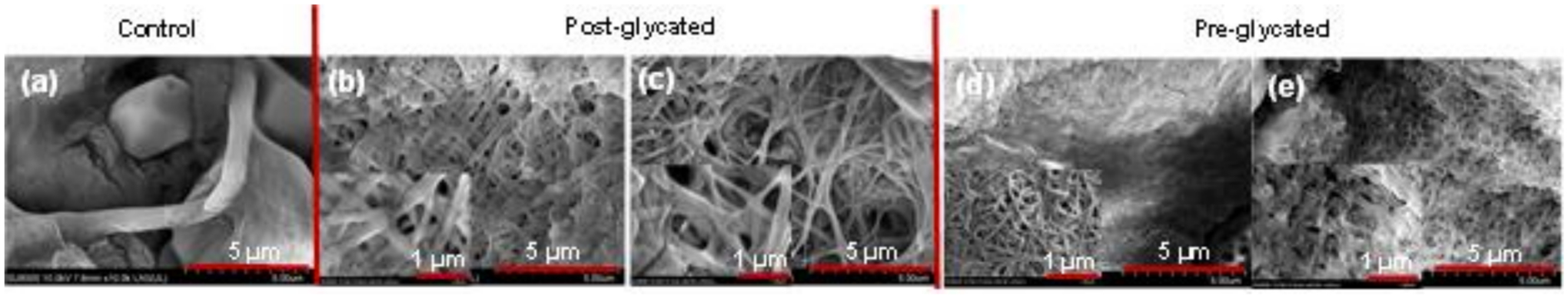
SEM images of (**a**) control, (**b**,**c**) post-glycated of (**b**) 1 mM, (**c**) 10 mM, and (**d**,**e**) pre-glycated of (**d**) 13 mM, and (**e**) 22 mM scaffolds.

As shown in Figure 4, SEM results show that collagen structural morphology was changed upon glycation with genipin. Both fiber size and network density were altered with increasing concentration of genipin, including a difference in structure depending on whether the scaffold was pre- or post-glycated (Figure 4).

The formation of denser fiber networks was an expected result when glycating the scaffolds. The increase in storage modulus as a result of glycation in the collagen scaffolds could offer more plasticity to the fibers, allowing them to stretch—and become thinner as a result—without breaking, as opposed to the control gels. The difference between pre-and post-glycation was consistent, as post-glycated gels retained the somewhat porous morphology of the control gels, while the gels pre-glycated with genipin were essentially crosslinked from within, resulting in the tight-knit “woven” morphology observed in the SEM images.

**Figure 5.**
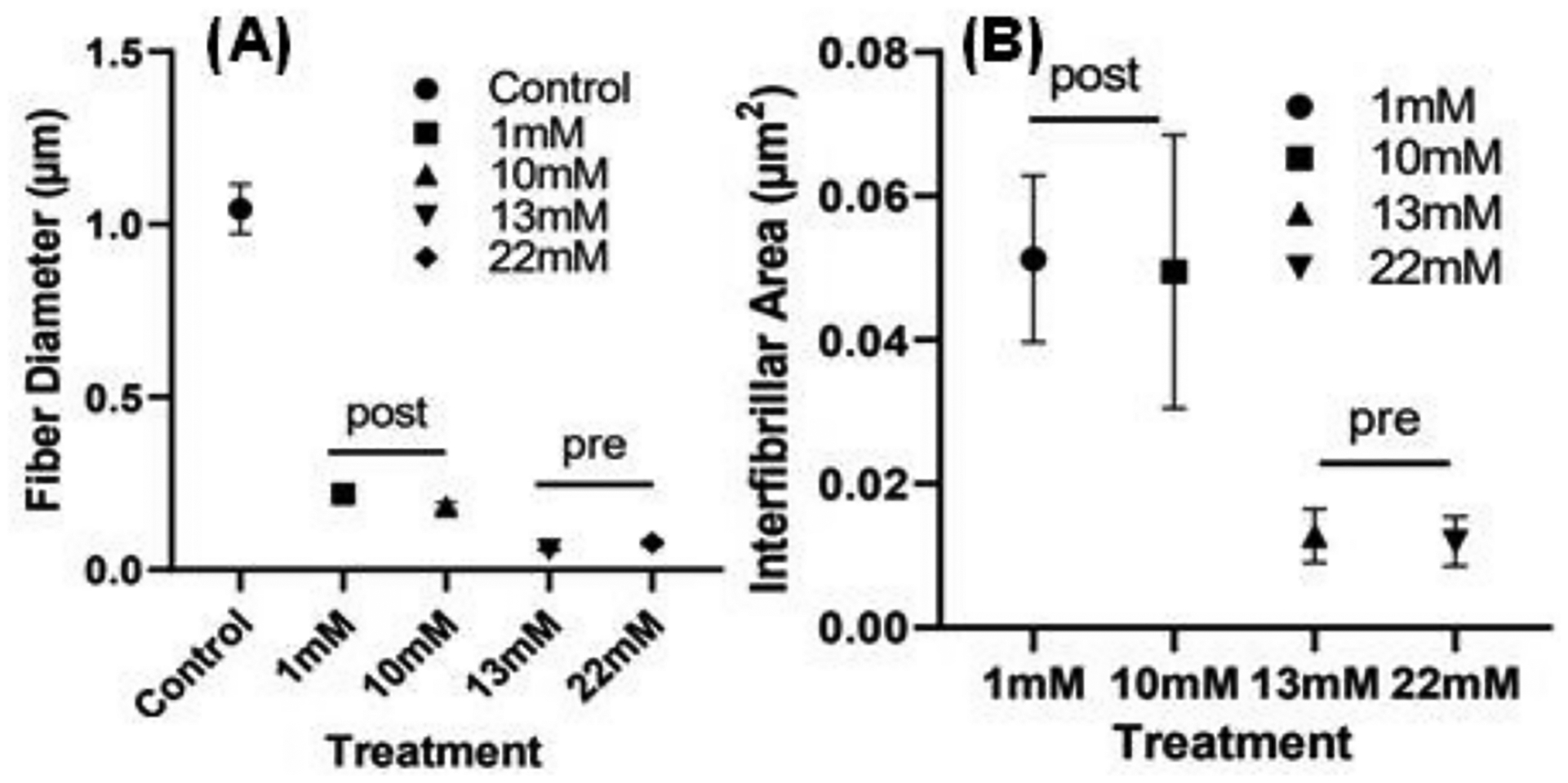
Comparison of (**A**) fiber diameter and (**B**) interfibrillar space among collagen scaffolds in pre- and post-glycated gels. Images analyses were conducted in replicates of 5 (*p* < 0.05).

The structural morphology of glycated scaffolds was analyzed in terms of fiber diameter and network density using ImageJ. Post-glycated gels had more porous scaffolds with larger interfibrillar spaces, while pre-glycated gels were more tightly woven (Figure 5). Regardless of glycation method, both pre- and post-glycated gels showed significantly smaller collagen fibers than the control gel.

### Cell proliferation and viability:

**Figure 6.**
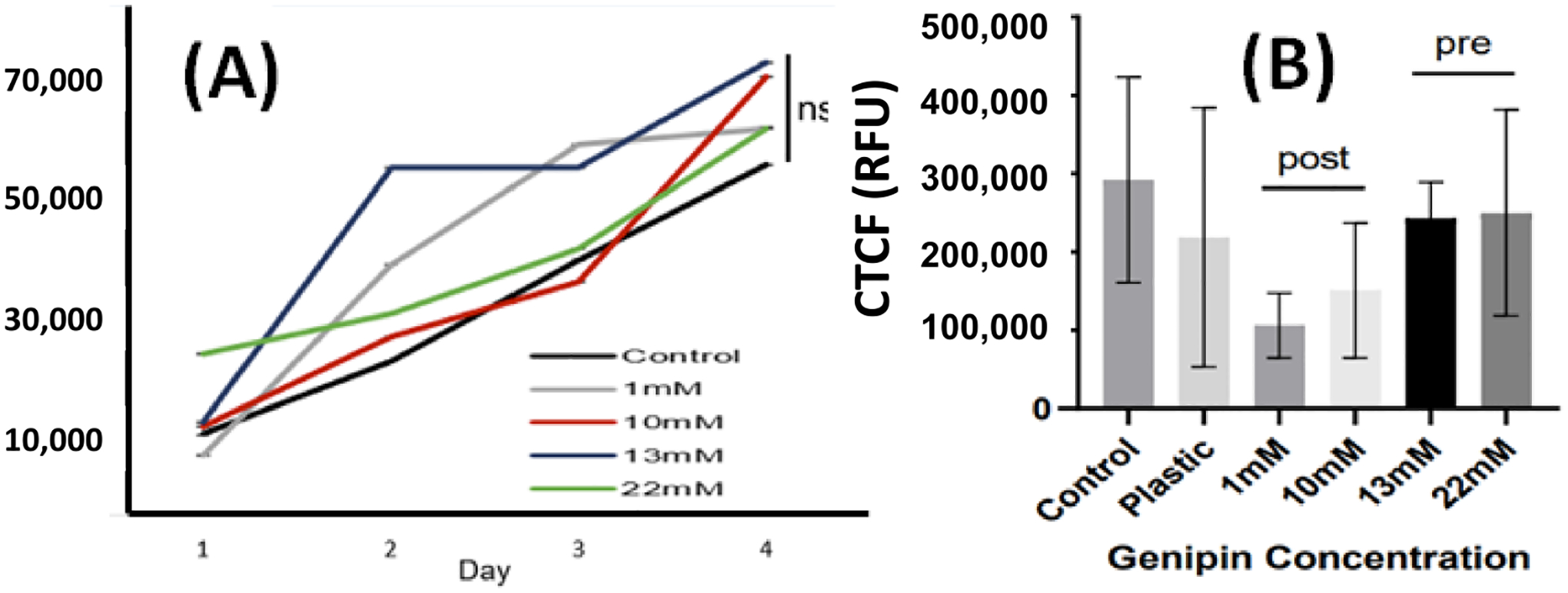
(**A**) Cell proliferation and (**B**) viability: cell counts were measured in three different areas and averaged over the course of 3 days (*p* = 0.9942), and live cells were stained with Calcein-AM and quantified in ImageJ (*p* = 0.2213).

Cell proliferation was one of the markers used to determine the cytotoxicity of the genipin-crosslinked collagen hydrogel. The cells were imaged in triplicate on each day of the 3 days of growth in the 24-well plate. The results show that there was no significant difference in total cell proliferation across gel treatments (Figure 6). According to ISO 10993-5, cell viability above 80% is considered as non-cytotoxic or biocompatible. As shown in Figure 6, pre-glycated collagen (higher genipin concentration) shows more biocompatibility then pre-glycated collagen.

Genipin was used as a noncytotoxic crosslinking agent which is safer than other common chemical crosslinkers such as glutaraldehyde [5]. However, most papers explored the actual cytotoxic nature of genipin, often using as small an amount as possible in order to optimize the balance between scaffold strength and cell viability. The effect of matrix hydrogel to host viable cells was not explored enough [8]. While this was the initial impression in this experiment upon glycation using genipin, the introduction of serial washes greatly improved cellular viability on scaffolds with higher concentrations of genipin.

Another method of introducing genipin to hydrogels was to dissolve the compound in DMSO [10]. The standard procedure was to dissolve genipin in a 1:3 ratio of DMSO to PBS. However, when this was attempted, it was found that the levels of DMSO were nearly 100 times the minimum levels for severe cytotoxicity. With regards to washing, most papers describe washing the glycated gels with deionized water; for example, Zhang et al., (2014) reported no significant difference in cell viability among scaffolds [7]. As others reported [7], we do not believe that the use of small amounts of genipin to cross-link collagen causes cytotoxicity. One possible reason for cytotoxicity would be the use of DMSO. We believe that washes with only dH2O were not sufficient to eliminate cytotoxicity from the glycated gels. Sundararaghavan et al., (2008) reported a seven-time decrease in the number of cells on their 10 mM genipin gel when compared to their control. However, their gels were unwashed, and instead of HUVECs, L929 fibroblasts were used [15].

**Figure 7.**
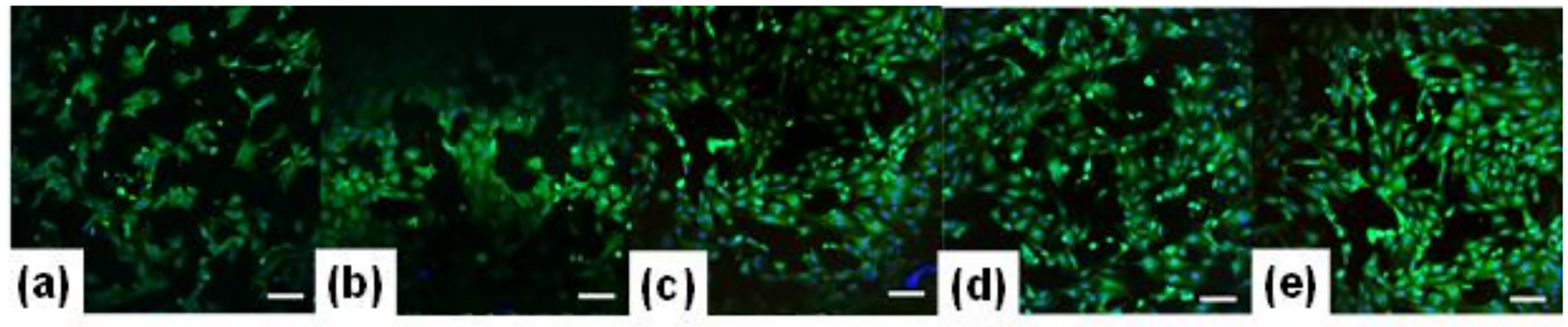
Claudin-5 expression (green) and Hoechst nuclei stain (blue). Letters (**a**–**e**) refer to control, 1 mM, 10 mM, 13 mM, and 22 mM concentration gels, respectively. Images taken at 10X. Scale bar = 100 um.

**Figure 8.**
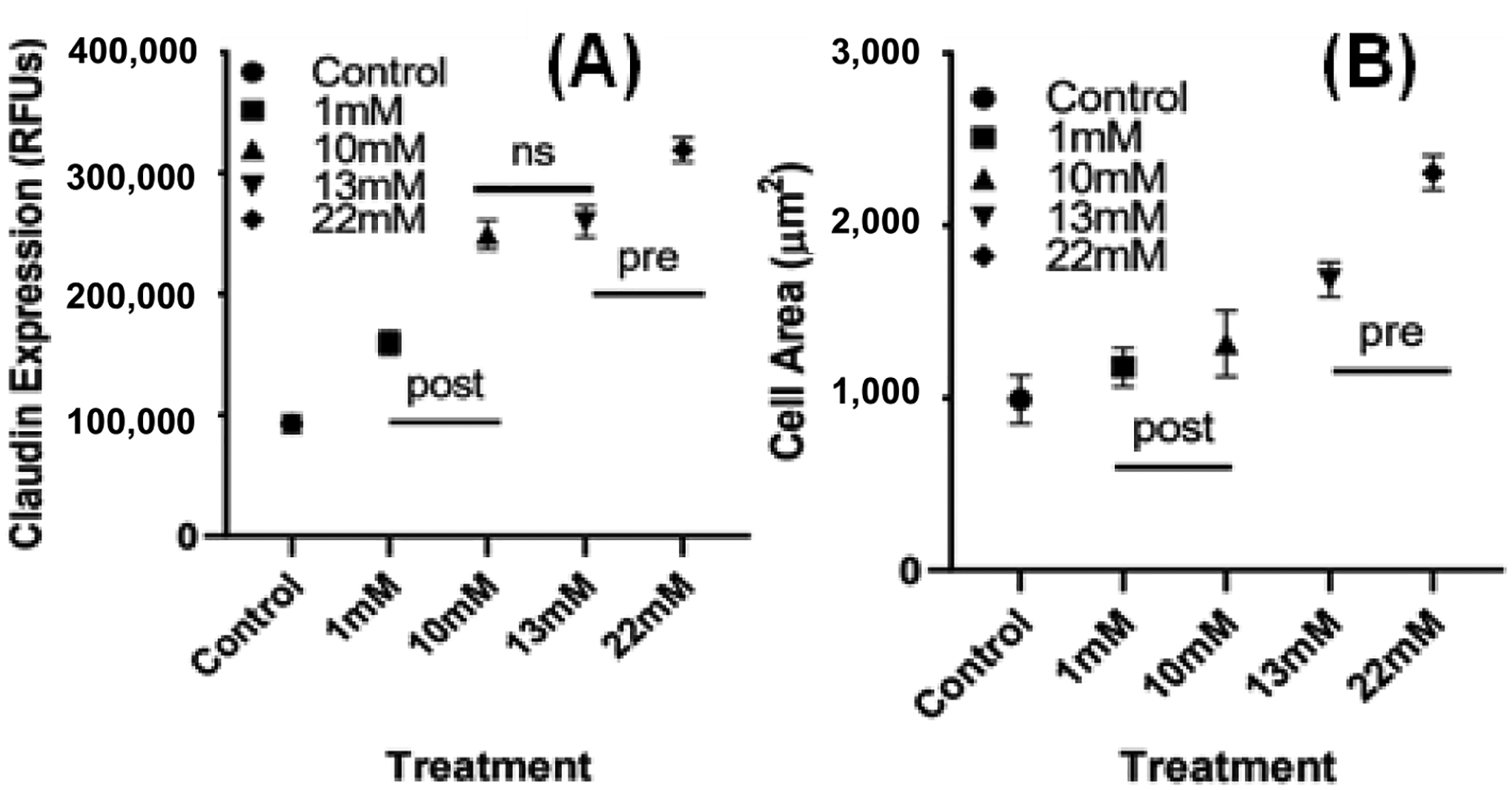
(**A**) Claudin-5 expression and (**B**) average cell area of HUVECS grown on control, pre- and post-glycated gels. Measurements were performed in triplicate on day 3 of HUVEC culture (*p* < 0.0001).

Claudin-5 was stained and imaged under confocal microscope to exam cell morphology (Figure 7). Average relative fluorescence among multiple cells per treatment was measured, and the expression of claudin-5 per scaffold was quantified. Results from Claudin-5 staining show where the intercellular junctional area is located, clearly showing the morphology of cells. It was found that glycation increased the cellular expression of claudin-5 when compared to the control. Even though the control sample (collagen only) shows higher viability (Figure 6), the cells in the control sample show more rounded morphology. Among the glycated scaffolds, the 10, 13, and 22 mM scaffolds had higher claudin-5 expression than the 1 mM gel, but the 10 mM and the 13 mM scaffolds had no significant difference in protein expression (Figure 8). Further cell spreading was analyzed using Image J. As described in Figure 8, pre-glycated gels had a higher degree of cell spreading than in post-glycated or control scaffolds. However, while the 22 mM gels had significantly higher levels of cell spreading from all other gels, the 13 mM scaffolds were found to have no significant difference from both the 1 and 10 mM gels.

### TEER:

**Figure 9.**
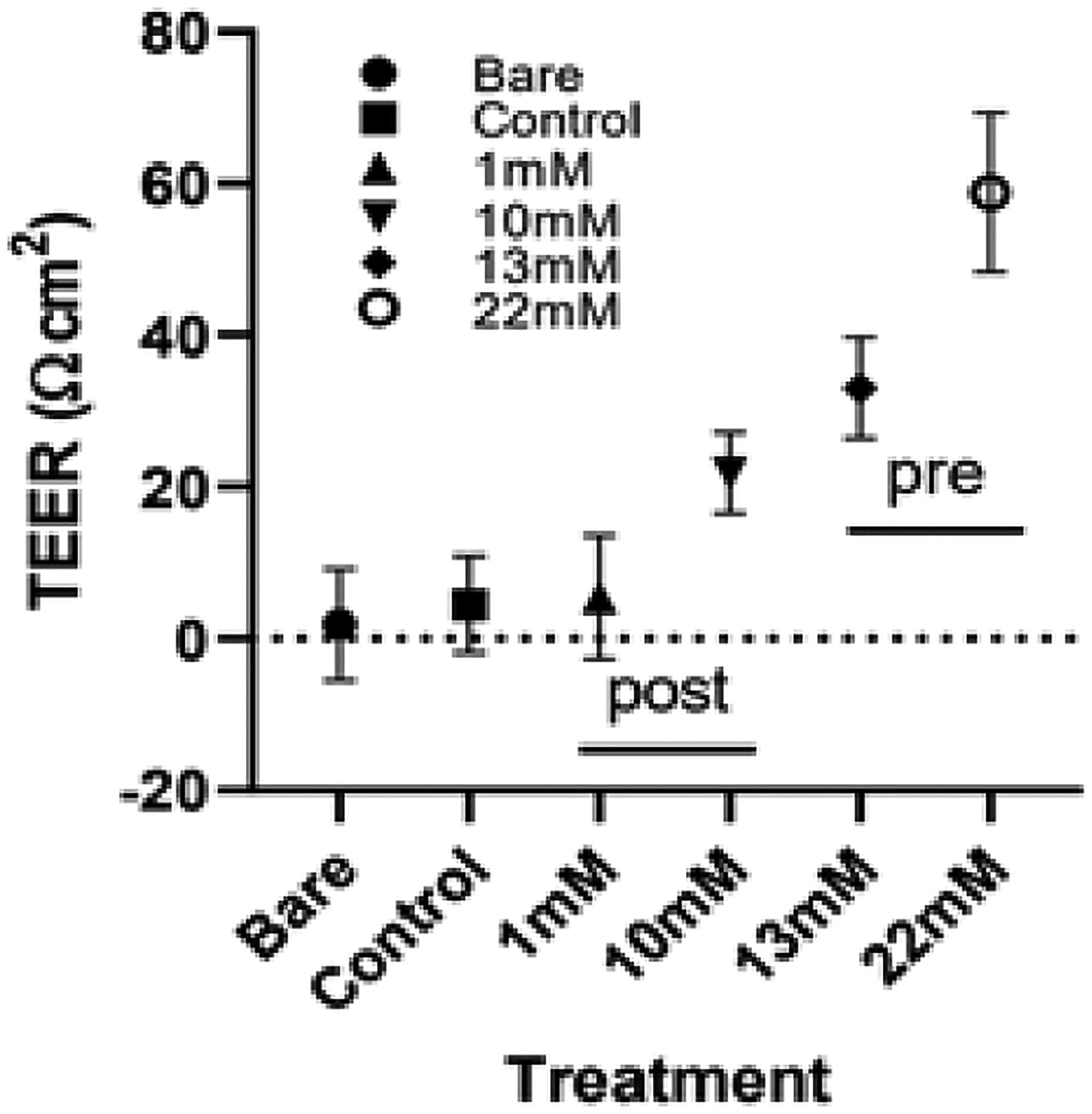
TEER measurements of cell cultures on varying substrates. Measurements were taken on day 3 of culture (*p* < 0.01).

As shown in Figure 9, the increase of genipin concentration increased TEER resistance. In particular, the 10, 13, and 22 mM scaffolds had significantly higher resistance than the bare Transwell membrane, as well as the control and 1 mM scaffolds.

TEER measurements resulted in an exponential-like curve in resistance at 3 days of growth with the increase of genipin concentration. There was variability in resistance measurements per sample, because collagen scaffolds are not flat surfaces, and the cells had not formed a perfect monolayer, thereby meaning that cell density varied in certain areas. This increase in TEER indicates the formation of a better endothelial barrier from gels grown on stiffer scaffolds, which is consistent with the other results of endothelial response observed in this experiment.

Generation of mechanical tunable matrix is critical for in vitro culture platform development such as tissue chip and organoid, in order to model diseases and disorders such as alzheimer’s disease; (1) alternative testing platform development to animal model; and (2) regenerative engineering such as wound healing.

## 4. Conclusions

This paper demonstrates that the glycating agent, genipin, allowed for the tunability of collagen hydrogels, ultimately producing scaffolds with a wide range of mechanical strength and structural morphology. These scaffolds were able to maintain endothelial cell viability, producing results of increased Claudin-5 protein expression, barrier integrity, and matrix adhesion. This lays the groundwork for future experiments, including, but not limited to, neurovascular unit tenability, drug testing, and drug delivery.
